# Human bone marrow-derived, pooled, allogeneic mesenchymal stromal cells manufactured from multiple donors at different times show comparable biological functions in vitro, and in vivo to repair limb ischemia

**DOI:** 10.1186/s13287-021-02330-9

**Published:** 2021-05-10

**Authors:** Charan Thej, Sudha Balasubramanian, Mathiyazhagan Rengasamy, Ankita Walvekar, Priyanka Swamynathan, Swathi Sundar Raj, Pradnya Shahani, Siddikuzzaman, Udaykumar Kolkundkar, Raviraja N. Seetharam, Pawan Kumar Gupta, Anish S. Majumdar

**Affiliations:** Stempeutics Research Pvt Ltd, 3rd Floor, Manipal Hospitals Whitefield Pvt. Ltd., #143, EPIP Industrial Area, K R Puram Hobli Bengaluru, India

**Keywords:** Pooled mesenchymal stromal cells, Stempeucel®, Multiple bone marrow aspirations, Angiogenesis, Hind limb ischemia

## Abstract

**Background:**

We have previously demonstrated that a pooled population of bone marrow-derived, allogeneic mesenchymal stromal cells (BMMSC), Stempeucel®-1, produced under good manufacturing practices (GMP) conditions, showed clinical efficacy and safety in patients suffering from critical limb ischemia (CLI) due to Buerger’s disease. While Stempeucel®-1 is currently used for CLI and other clinical indications, we wanted to ensure that the product’s continuity is addressed by developing and characterizing a second generation of pooled product (Stempeucel®-1A), manufactured identically from second BM aspirates of the same three donors after a 2-year interval.

**Methods:**

The two versions of Stempeucel® were manufactured and subjected to gene and protein expression analysis. The nature of various growth factors/cytokines secreted and immunomodulatory activity of these two cell populations were compared directly by various in vitro assays. The preclinical efficacy of these two cell types was compared in an experimental model of hind limb ischemia (HLI) in BALB/c nude mice. The reversal of ischemia, blood flow, and muscle regeneration were determined by functional scoring, laser Doppler imaging, and immunohistochemical analyses.

**Results:**

Qualitative and quantitative analyses of genes and proteins involved in promoting angiogenic activity and immune regulatory functions revealed high levels of correlation between Stempeucel®-1 and Stempeucel®-1A cell populations. Moreover, intramuscular (i.m) administration of these two cell products in the ischemic limbs of BALB/c nude mice showed significant repair (≥ 70%) of toe and foot necrosis, leading to improved ambulatory function and limb salvage. Furthermore, a biodistribution kinetics study showed that Stempeucel®-1 was mostly localized in the ischemic muscles of mice for a significantly longer time compared to normal muscles, thus playing an essential role in modulating and reversing HLI damage.

**Conclusions:**

This study shows that with a reproducible manufacturing procedure, it is possible to generate large numbers of pooled mesenchymal stromal cells from human bone marrow samples to establish product equivalence. We conclude from these results that, for the first time, two pooled, allogeneic BMMSC products can be repeatedly manufactured at different time intervals using a two-tier cell banking process with robust and comparable angiogenic properties to treat ischemic diseases.

## Background

In recent years, therapeutic angiogenesis has been achieved by delivering proangiogenic factors either by gene delivery or cell therapy [1]. The cell-based approach has gained significant momentum in the management of peripheral arterial disease (PAD). A variety of cells are known to possess angiogenic functions such as CD34^+^ hematopoietic stem cells (HSC) [2], bone marrow mononuclear cells (BMMNC) [3], endothelial progenitor cells (EPC) [4], mesenchymal progenitor cells (MPC) [5], and mesenchymal stromal cells (MSCs) [6]. So far, BMMNCs, EPCs, and MSCs have been shown to ameliorate or reduce the effect of limb ischemia by improving amputation-free survival, ankle-brachial index (ABI), transcutaneous oxygen pressure (TcPO_2_), ulcer healing, and reduced rest pain to variable extents in no-option critical limb ischemia (CLI) patients [3]. Among the tissue sources, bone marrow-derived MSC (BMMSC) expansion method is well established in various laboratories and is thoroughly characterized as a result of which BMMSC are the principal source of MSCs for the majority of preclinical and clinical studies conducted so far [7, 8]. In addition, positive efficacy data have also been observed with adipose tissue-derived MSC (ADSC), placenta-derived MSC (PDMSC), Wharton’s jelly-derived MSCs (WJ-MSC), and MSCs differentiated from pluripotent stem cells for various disease indications, including CLI [9]. MSCs obtained from various tissue sources, including BMMSC, are usually isolated by their plastic adherence property and are generally characterized following the recommendations of the International Society of Cell and Gene Therapy [10], which are expanded in large-scale culture for clinical use. While initial success of MSC administration for the treatment of CLI was demonstrated in various preclinical and early and late phase clinical trials, consistent production and robust demonstration of efficacy between different manufacturing batches of cells have been recognized as a daunting task for these cell products to take it beyond a certain stage of development [11]. Demonstration of consistent characteristics of a cell therapy product in terms of its in vitro functional attributes and preclinical efficacy and safety in a suitable animal model of limb ischemia has been in demand for the long-term sustainability of the product. Such studies are key to determine the disease-modifying ability of these MSCs successfully.

Our first generation of BMMSC product, which was designated as Stempeucel®-1, is a human bone marrow-derived, cultured, pooled, allogeneic BMMSC product obtained from the bone marrow (BM) aspirates of several consenting donors [12, 13]. The pooling of bone marrow-derived MSCs from three individual donors would presumably eliminate variability seen with individual BMMSC donors and generate a consistent product. Stempeucel®-1 has been manufactured from BMMSCs of three healthy consenting donors using a patented two-tier cell banking technology (US8956862). Subsequently, we also published the growth condition, phenotypic and functional characterization, gene expression, differentiation, angiogenic potency, and cryopreservation of individual and pooled BMMSCs [14–16]. In fact, we have demonstrated that Stempeucel®-1 secretes a wide array of proangiogenic GFs such as vascular endothelial growth factor (VEGF), angiopoietin-1 (Ang-1), stromal-derived factor 1α (SDF-1α), interleukin-8 (IL-8), IL-6, hepatocyte growth factor (HGF), and transforming growth factor β1 (TGFβ1) consistently in all batches of Stempeucel®-1 [17]. Among these factors, VEGF was found to be secreted in a reproducible range in several large-scale expanded batches of Stempeucel®-1. Further, we elucidated the mechanism of action (MoA) of Stempeucel®-1 population to establish the functional implications of VEGF secretion to directly demonstrate the angiogenic properties of Stempeucel®, which is mediated predominantly by VEGF in vitro [17]. The therapeutic angiogenic potential of Stempeucel®-1 was established in a well-accepted preclinical model with the A-strip method of unilateral limb ischemia in athymic BALB/c nude (OlaHsd-Fox1nu) mice [13, 18]. We found that intramuscular (i.m) administration of Stempeucel® ameliorated limb necrosis and improved limb function in 28 days [7, 12]. We also published the results of our phase II, open-label, non-randomized, dose-finding study to evaluate the efficacy of Stempeucel® in CLI due to Buerger’s disease [13].

So far, Stempeucel®-1 was used to carry out clinical trials for CLI and other disease indications. To further expand the lifespan of an efficacious cell therapy product, we manufactured the second generation of Stempeucel® known as Stempeucel®-1A from the same set of donors at a different time interval and compared its cellular and molecular characteristics with those of Stempeucel®-1. In this article, we show the similarities between the two versions of Stempeucel® to determine some critical attributes necessary for demonstrating the efficacy and safety in the limb ischemia model. The collective data presented in this manuscript validates our robust manufacturing protocol and shows that both the products manufactured from the same donors at an interval of 2 years possess highly comparable characteristics, including angiogenic function, both in vitro and in vivo.

## Methods

### Bone marrow aspiration, preparation of master cell banks, working cell banks, and Stempeucel® from two different aspirations from the same donors at different times

The methodology of isolating BM, preparation of master cell banks (MCB), working cell banks (WCB), and preparation Stempeucel® has been published previously [13, 15] and has also been patented (US8956862). Briefly, the MCB comprises of BMMSCs derived from individual donors and the WCB comprises a pooled population of BMMSCs. The working cell bank-1 (WCB-1) was prepared from the first BM aspirates from three donors, and subsequently, the WCB-1A was prepared from the BM aspirated obtained from the same three donors 2 years apart. Stempeucel®-1 is manufactured from WCB-1, and similarly, Stempeucel®-1A is manufactured from WCB-1A batch. Stempeucel®-1 is formulated and cryopreserved in 85% Plasmalyte A (Baxter, Illinois, USA), 10% dimethyl sulfoxide (DMSO) (Sigma-Aldrich, Missouri, USA), and 5% human serum albumin (HSA) (Sigma-Aldrich, Missouri, USA), and Stempeucel®-1A is formulated in CS5 (CryoStor 5, Biolife Solutions, Washington, USA). In this study, we evaluated the comparability of the Stempeucel®-1 and Stempeucel®-1A manufactured from two different WCB batches 1 and 1A and cryopreserved in plasmalyte A and CS5 formulations respectively. The basic criteria for the characterization of Stempeucel®-1 and Stempeucel®-1A are given in supplementary Table 1. The techniques used to characterize Stempeucel® have been published previously [14, 15].

### Gene expression profiling by microarray and analysis of Stempeucel®-1, Stempeucel®-1A, and human foreskin fibroblasts (HFF)

Total RNA was isolated using the RNeasy Micro kit (Qiagen, Hilden, Germany) as per the manufacturer’s protocol. The quality of the extracted RNA was checked using Nanodrop Spectrophotometer (Thermo Fisher Scientific, Waltham, USA). The OD 260/280 ratio was > 1.9 and OD 260/230 was > 1.8 for all samples. Further, RNA integrity was assessed using 2100 Bioanalyzer (Agilent Technologies Inc., Santa Clara, USA) and RNA 6000 Nano kit. The RIN number of all RNA samples was > 8.0. For each sample, 250 ng of total RNA was amplified and labeled using the Ambion WT Expression Kit (Thermo Fisher Scientific, Waltham, USA) and Affymetrix GeneChip WT terminal labelling kit (Affymetrix, Santa Clara, USA), respectively, according to the protocol provided by the supplier. The labeled second cycle cDNA was processed for further analysis using a previously published method [19]. Unsupervised hierarchical clustering of differentially expressed genes was done using a Euclidian algorithm with Centroid linkage rule to identify gene clusters whose expression levels are significantly reproduced across the replicates.

### Immunophenotypic characterization by flow cytometry

The cells were incubated with the below-mentioned antibodies for half an hour at room temperature, following which the samples were analyzed using the FACS DIVA and WinMDI 2.9 software. The expression levels of CD73, CD105, CD44, CD166, CD90, HLA-ABC, CD34, CD45, HLA-DR, CD40, CD80, and CD86 were analyzed. The details and concentrations of the antibodies used are provided in supplementary Table 1.

### Assessment *of* in vitro immunomodulatory properties of Stempeucel®-1 and Stempeucel®-1A

For immunosuppression assays, a one-way mixed lymphocyte reaction (MLR) assay was performed at a ratio of 1:2.5, 1:5, and 1:10 (peripheral blood mononuclear cells (PBMSC): MSC) as described before [20]. For inflammatory cytokine priming, the MSC growth medium was replaced with 10 ml/flask of complete medium supplemented with interferon-γ (IFN-γ) (10 ng/ml) (Thermo Fisher Scientific, Waltham, USA) and tumor necrosis factor α (TNFα) (15 ng/ml) (Thermo Fisher Scientific, Waltham, USA). After 40 h of priming, cells were used for the MLR assay. Cell proliferation was measured using a fluorimetric immunoassay kit (Millipore-Sigma, Burlington, USA) to quantify Bromodeoxyuridine (BrdU) incorporation, according to the manufacturer’s instructions. A one-way MLR cultured in the absence of MSCs would be considered as the 100% proliferation control. All treatments were performed in triplicates.

### Enzyme-linked immunosorbent assay

Human angiogenic cytokines, VEGF, Ang-1, SDF-1α, IL-6, IL-8, HGF, and TGFβ1 in the conditioned media (CM), which was collected at the 72-h time point as described before [17], were estimated using Enzyme-Linked Immunosorbent Assay (ELISA) kits (R&D Systems, Minneapolis, USA) according to the manufacturer’s directions. The samples were assayed in duplicates. Error bars are expressed as mean value ± SEM.

### In vitro angiogenic activity assessment of Stempeucel®-1 and Stempeucel®-1A

The CM collected at the 72-h time point from Stempeucel®-1 and Stempeucel®-1A was used to evaluate three in vitro angiogenic functional assays using human umbilical vein endothelial cells (HUVECs). The HUVEC migration, proliferation, and tube formation assays were performed as described in our previous publication [17].

### Animal experiments

#### Animal model and cell injection procedure

Unilateral hind limb ischemia was established in 10–12-week-old BALB/c nude (OlaHsd-Fox1nu) mice as described before [13]. Stempeucel®-1 and Stempeucel®-1A were administered at a dose of 5 × 10^6^ cells in 50 μl of Plasmalyte A as determined to be the maximum effective dose for Stempeucel®-1 and published previously [7], or vehicle (Plasmalyte A) was administered by i.m injection using a 26G needle, around the ligation site, at five different places (approximately 10 μl at each site), following 2-5 h post-induction of limb ischemia to enable animals to recover from the trauma of surgery and anesthesia. All the animals were observed for 28 days at regular intervals.

### Limb necrosis and functional scoring

The therapeutic effect of Stempeucel® in ameliorating the progression of tissue necrosis was assessed by examining the number of toes necrosed following ligation. Necrotic scoring was performed on days 0, 7, 14 & 28. Similarly, clinical and functional outcome following Stempeucel® treatment was assessed by Tarlov score, ischemic score, and ambulatory scores as described before [21].

### Blood flow measurement by laser Doppler imaging

The rate of blood perfusion was measured for both ischemic and normal limb for both cell and vehicle-treated groups using laser Doppler imaging system (LDI2, Moor Instruments, UK) at the Department of Pharmacology, PSG College of Pharmacy, Coimbatore, India. The blood flow measurements were expressed as a ratio of the flow in the ischemic limb versus the normal limb.

### DiI Labelling of Stempeucel®-1 followed by intramuscular administration for biodistribution analysis

To examine the persistence of bone marrow-derived MSC in the hindlimb ischemia model in BALB/c nude mice, the cells were stained with Vybrant™ CM-DiI Cell-Labeling Solution (Thermo Fisher Scientific, Waltham, USA) as described before [22], prior to injecting the cells into the animals. Five million viable cells were resuspended in 50 μl PlasmaLyte A and injected i.m in both ischemic and normal limbs. Evaluation of signal intensity from CM-DiI-labeled cells was performed after cell injection on days 1, 3, 6, 11, 14, 21, and 28 using the In Vivo Imaging System (IVIS) Xtreme Imaging System (Bruker, Massachusetts, USA). The images were quantified using Carestream molecular imaging software. As per the IVIS Xtreme Imaging System guidelines, all the animals were imaged before the cell injection to normalize autofluorescence. Average pixel intensity from each group was calculated from the individual animal’s pixel area intensity.

### Histopathological evaluation

The therapeutic effect of Stempeucel® was also evaluated based on histological analysis of the various muscle sections (adductor, soleus, gastrocnemius, and semimembranosus/gracilus), stained with H & E. The stained muscle sections were scored for muscle degeneration, inflammation, and muscle necrosis from five separate fields in four distinct areas. Total numbers of each incident of degeneration, inflammation, and necrosis were calculated from each group. In animals that presented auto-amputation, the muscles were not collected, and in such cases, the maximum histological scoring of 5 (severe) was given for the degeneration, inflammation, and muscle necrosis. Muscle fiber area was quantified using QWin Software (Leica Biosystems, Wetzlar, Germany). Immunohistochemical analysis was performed on paraffin-embedded muscle tissue sections (5 μm thickness) of mice using anti-mouse CD31 antibody (Cat no. SAB4502167; Sigma-Aldrich, Missouri, USA), anti-human nuclear antigen (HNA) (Cat no. ab191181; Abcam, Cambridge, UK), and anti-human VEGF antibody (Cat no. ab46154; Abcam, Cambridge, UK). HRP-conjugated secondary antibodies, anti-rabbit IgG (EnVision+, Agilent technologies, CA, USA), goat anti-mouse IgG (Cat no.205719; Abcam, Cambridge, UK), and goat anti-rabbit IgG (Cat no. 205718; Abcam, Cambridge, UK) were used for CD31, HNA, and VEGF staining respectively. The immunoreactive products were visualized as described before [23]. The evaluation was carried out by blinded pathologists who were not aware to which group the animals were allocated.

### Statistical analysis

The results were represented as mean ± SD or SEM. All the groups were compared by one-way ANOVA followed by Dunnett’s multiple comparisons using GraphPad Prism 5 software. *P* < 0.05 was considered a significant change.

## Results

### Consistency of gene expression pattern between various batches of Stempeucel®-1 and Stempeucel®-1A

**Fig. 1:**
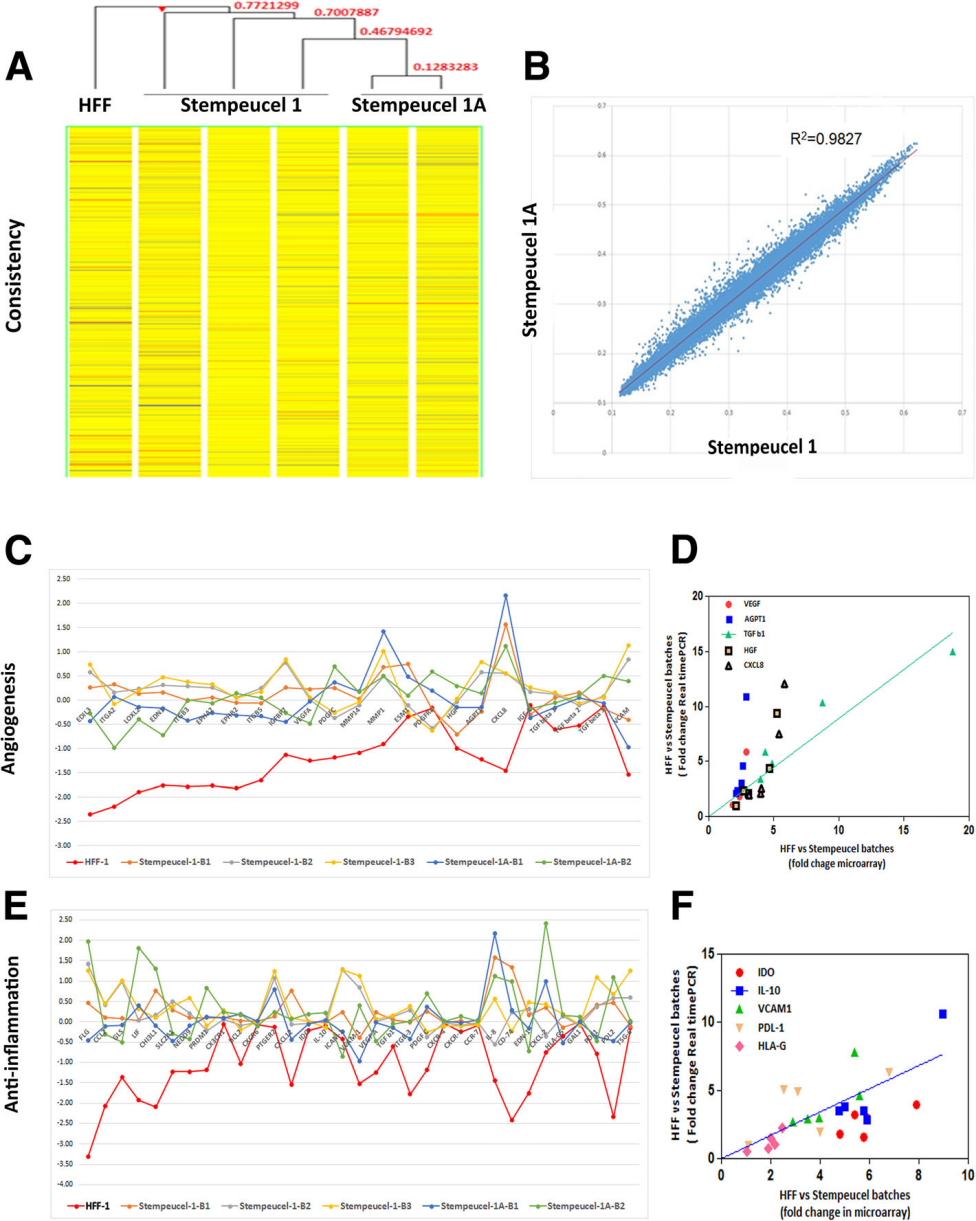
Gene expression profiling. **a** Unsupervised hierarchical clustering/condition tree of samples, HFF-1, three batches of Stempeucel®-1 and two batches of Stempeucel®-1A together based on the similarity of their expression profiles. It is evident from the condition tree based on the distance that out of 24,498 genes, an average of ≥ 77% of the global profiles of all the Stempeucel® products is similar compared to the HFF cells indicating a passage-independent similarity in global gene expression profiles. ~ 20% of the genes (4924 out of 24,498 genes) analyzed were differentially expressed between Stempeucel®-1 and Stempeucel®-1A**.** In total, ~ 3% of the genes (722 out of 24,498 genes analyzed) were significantly differentially expressed with a fold change of ≥ 2. **b** Analysis of genes expressed by Stempeucel®-1 (*X*-axis) and Stempeucel®-1A (Y-axis) with a significantly high correlation of *R*^2^ = 0.9827 in a scatter plot. **c** Comparative evaluation of twenty-three angiogenic genes in HFF-1 cell line, three batches of Stempeucel®-1, and two batches of Stempeucel®-1A. **d** Real-time PCR analysis of five selected angiogenic genes co-expressed across the Stempeucel® batches compared to HFF-1; VEGF—*P = 0.03, AGPT1—*P = 0.04, TGF b1—*P = 0.01, HGF—*P* = 0.03, and CXCL8—*P = 0.02. **e** Comparative evaluation of thirty-four anti-inflammation genes in HFF-1, three batches of Stempeucel®-1 and two batches of Stempeucel®-1A. **f** Real-time PCR analysis of five selected anti-inflammatory genes co-expressed across Stempeucel® batches compared to HFF-1; IDO—**P = 0.001, IL-10—**P = 0.004, VCAM1 **P = 0.002, PDL1—**P = 0.009, and HLA-G—**P = 0.002

Global gene expression profile analysis was used to assess the consistency and comparability of the pooled Stempeucel® products obtained from two batches each of WCB 1 and 1A that were used to manufacture the pooled BMMSC products. Three GMP large-scale batches of Stempeucel®-1 and two Stempeucel®-1A batches were analyzed after evaluating the quality control parameters. We have performed an unsupervised hierarchical clustering of samples based on the similarity of their gene expression profiles (Fig. 1). It is evident from the condition tree-based analysis on the distance that out of a total of 24,498 genes analyzed, an average of ≥ 77% of the global gene profiles across all the batches manufactured from two different WCBs is similar, indicating a passage-independent similarity in the global gene expression profiles of the two pooled BMMSC populations. Gene clustering also clearly showed comparability between the expression profiles of Stempeucel®-1 and Stempeucel®-1A batches while each batch-related products remain as distinct clusters, and the distance between them is non-significant (Fig. 1a). We observed ~ 20% (4924 of 24,498) of the genes were differentially expressed between Stempeucel®-1 and Stempeucel®-1A; however, the difference in magnitude was not significant. While performing further analysis, we found that ~ 3% (722 of 24,498) of genes were expressed significantly, with a fold change of ≥ 2. However, these genes were responsible for biological processes (e.g., AGAP, ACP5, UCHL1), metabolic processes mainly related to mitochondrial function (e.g., PID1, PMAIP1), lipid metabolism (e.g., PID1, PTGDR), ECM synthesis (e.g., CNTNAP3, LAMA1), and cell response to TGFβ-linked morphogenesis functions (e.g., AHI1, LAMA1, MEF2C, ZEB1, and CXCR1). In a scatter plot analysis, we further observed a significantly higher and stronger correlation with an *R*^2^ value of 0.9827 between Stempeucel®-1 and Stempeucel®-1A batches (Fig. 1b) as compared to that of Stempeucel® and HFF, for which the *R*^2^ value was observed to be 0.9290 (data not shown). These results demonstrate that Stempeucel® batches of 1 and 1A profiled globally together for most of the genes analyzed.

Next, we analyzed the expression profiles of essential 23 angiogenic genes (Fig. 1c; Supplementary Table 2) and 34 anti-inflammation genes (Fig. 1e; Supplementary Table 3), known to play important roles in the neoangiogenic and immunoregulatory properties of BMMSC that are imperative in allogeneic stem cell-based therapy (Fig. 1c, e; supplementary Tables 2 and 3). The average gene expression values of both angiogenic and anti-inflammatory genes were significantly consistent across the five Stempeucel® batches (3 of Stempeucel®-1 and 2 of Stempeucel®-1A). In contrast, most of the genes were expressed at a lower level in the human foreskin fibroblast (HFF) cell line (Fig. 1c, e). The microarray data were validated by quantitative real-time PCR assay for key angiogenic genes VEGF, AGPT1, TGFβ1, HGF, and CXCL8/IL-8 and significant correlation was observed for all the five genes between Stempeucel®-1 and Stempeucel®-1A (Fig. 1d). Similarly, we also obtained a significant correlation for anti-inflammatory genes like indoleamine 2,3-dioxygenase (IDO), IL-10, vascular cell adhesion molecule (VCAM1), programmed death ligand 1 (PDL1), and HLA-G between the two Stempeucel® versions (Fig. 1f). The data suggest that both the pooled allogeneic BMMSC populations exhibit a comparable level of gene expression of key angiogenic and immunomodulatory factors by microarray, further confirmed by RT-PCR analyses.

### Assessment of in vitro immunosuppression of alloreactive MLRs by Stempeucel®-1 and Stempeucel®-1A

**Fig. 2:**
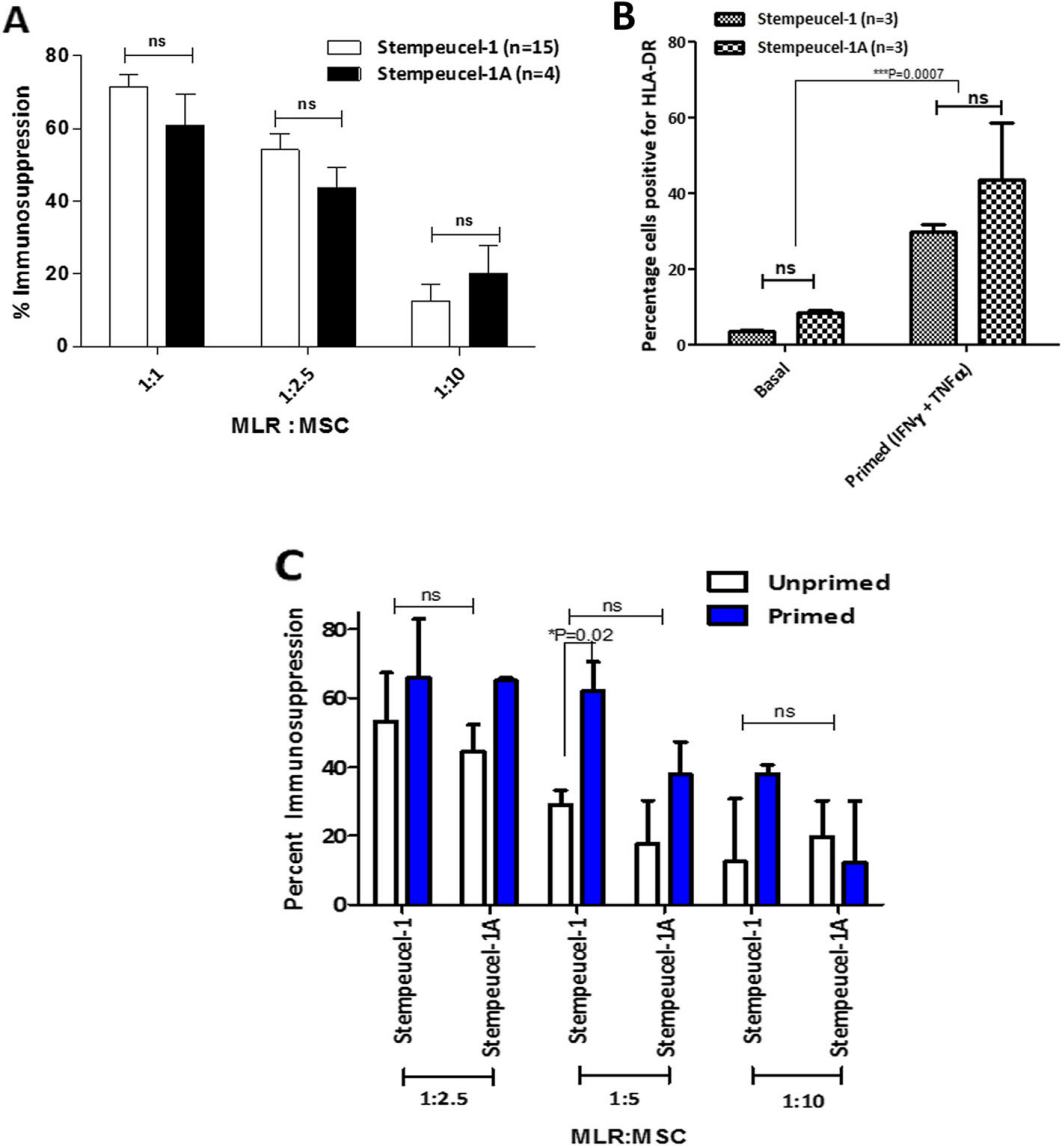
In vitro immunosuppression of alloreactive MLRs by Stempeucel®-1 and Stempeucel®-1A. **a** Suppression of MLR by mitomycin C-arrested Stempeucel®-1 (*n* = 15) and Stempeucel®-1A (*n* = 4) at three MLR: MSC ratios of 1:1, 1:2.5, and 1:10; MLR proliferation and suppression was measured by BrdU incorporation measurement as described in the “Methods” section. **b** Percentage of cells positive for HLA-DR in Stempeucel®-1 (*n* = 3) and Stempeucel®-1A (*n* = 3) cells at the basal level and after priming with TNFα and IFN-γ showed significant difference (***P = 0.0007). **c** Immunosuppression activity was retained in both Stempeucel®-1 and Stempeucel®-1A upon inflammatory priming at all three ratios of IFN-γ and TNFα primed, high HLA-DR expressing Stempeucel®-1 and Stempeucel®-1A cells did not reduce their immunosuppression capacity and a trend towards higher immunosuppression by primed cells was seen compared to unprimed controls. Graphs represent mean ± SD values; ns – not significant

Immunosuppressive activity of the two versions of Stempeucel® (in two different formulations) was compared by measuring the immunosuppressive activity of Stempeucel® against allogeneic PBMC responder cells in an in vitro MLR assay. The immunosuppressive activity of Stempeucel® was measured from fifteen batches of Stempeucel®-1 and four batches of Stempeucel®-1A, at different stimulator to responder ratios (Fig. 2a). Greater than 60% inhibition of T cell proliferation was observed with Stempeucel®-1, while Stempeucel®-1A showed > 70% inhibition by mixed lymphocyte reaction (MLR) assay (Fig. 2a). However, the difference in the inhibition of T cell proliferation induced by both versions of Stempeucel® was not found to be significant. The suppressive activity was titrable with decreasing number of MSC; however, moderate to high immunosuppressive activity was observed at 1:2.5, which further titrated out at 1:10 dilution (Fig. 2a). The difference in the MLR activity between the two Stempeucel® products was not found to be significant.

Although both pooled populations of Stempeucel® expressed low levels of human leukocyte antigen DR (HLA-DR), priming of these cells using a cocktail of IFN-γ and TNFα resulted in significant upregulation of HLA-DR (Fig. 2b). Furthermore, inflammatory cytokines primed BMMSC showed a concomitant increase in their ability to suppress allogeneic MLR, a finding that has been reported in the literature before [24]. However, similar to their unprimed counterparts, no significant difference was observed between Stempeucel®-1 and Stempeucel®-1A (Fig. 2c), thus emphasizing the immunological comparability between the two products.

### Angiogenic properties of Stempeucel®-1 and Stempeucel®-1A products

**Fig. 3:**
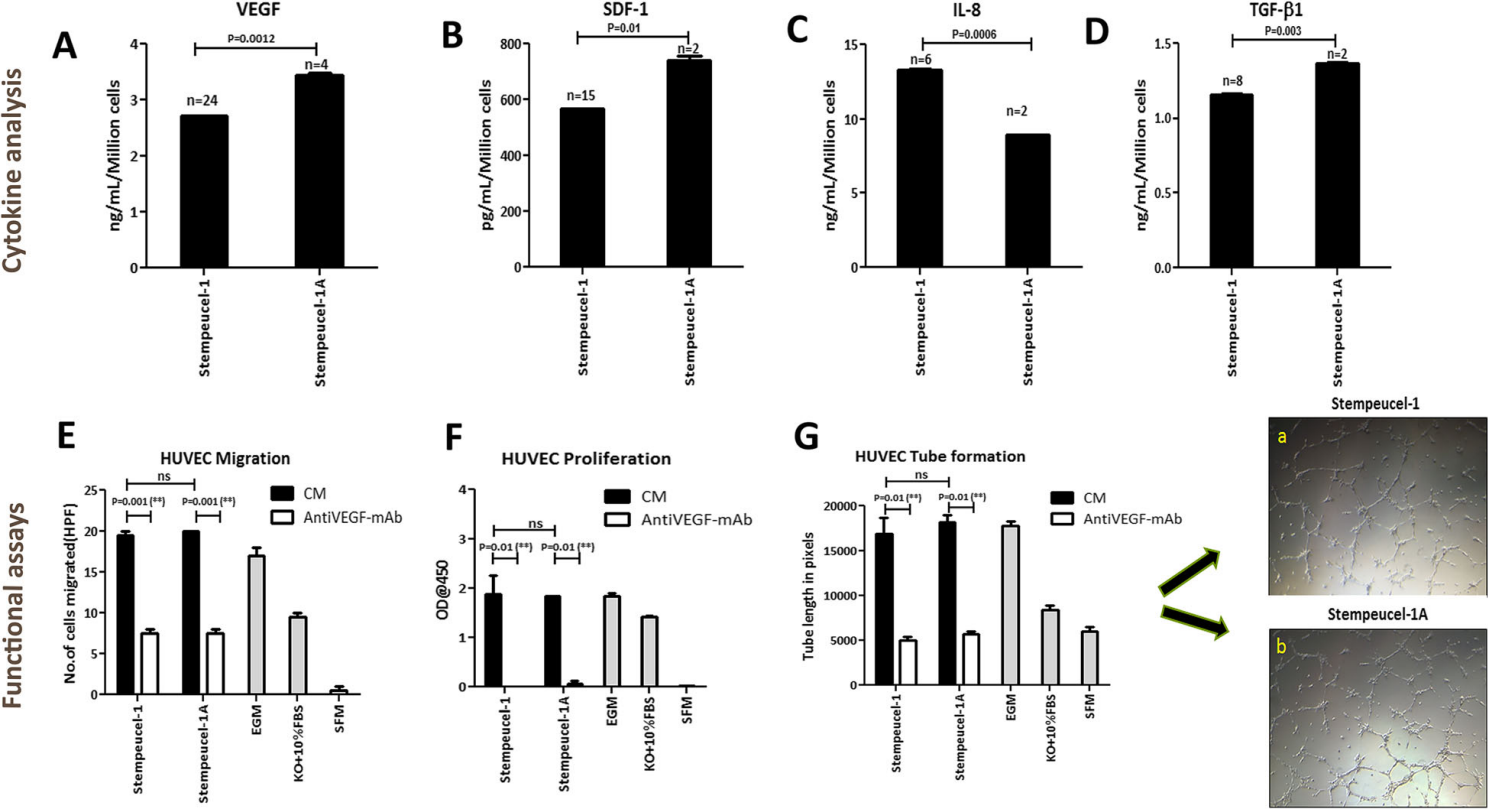
Quantification of angiogenic growth factors. **a** VEGF (A), **b** SDF-1α, **c** IL-8, and **d** TGFβ1 levels in the CM collected at the 72 h time point from Stempeucel®-1 and Stempeucel®-1A batches. Significant variation in the levels was observed between the two Stempeucel® versions, VEGF—**P = 0.001; SDF-1α—*P = 0.01; IL-8—***P = 0.0006; TGFβ1—**P = 0.003. **e** The CMs derived from both Stempeucel®-1 and Stempeucel®-1A promoted HUVEC migration equally. Addition of anti-VEGF mAb in the CMs significantly inhibited HUVEC migration (Stempeucel®-1 = 61.6%; Stempeucel®-1A = 61.6%). **f** Equivalent HUVEC proliferation was observed with both Stempeucel®-derived CM. Significant inhibition in HUVEC proliferation was observed upon neutralizing VEGF in the CMs (Stempeucel®-1 = 97.9, Stempeucel®-1A = 100%). **g** No significant difference in the HUVEC tube formation efficiency was observed between the CMs derived from Stempeucel®-1 and Stempeucel®-1A and similar inhibition (Stempeucel®-1 = 69.6%, Stempeucel®-1A = 65.1%) was observed between the two CMs in the presence of anti-VEGF mAb; a, b Similar HUVEC tube forming efficiency was observed with the CMs from both Stempeucel®-1 and Stempeucel®-1A. EGM—endothelial growth medium control; KO + 10%FBS – DMEM-KO basal medium plus 10% FBS control; SFM: serum-free DMEM-KO medium. Graphs represent mean ± SEM values

First, we quantified the levels of the key angiogenic factor VEGF in the CM from both versions of Stempeucel® at 72 h (Fig. 3a). The levels of VEGF secreted by Stempeucel®-1 batches (*n* = 24) was found to be 2.8 ± 1.0 ng/mL/10^6^ cells and for Stempeucel®-1A (*n* = 4), it was 3.45 ± 0.03 ng/mL/10^6^ cells. We observed that both Stempeucel®-1 and Stempeucel®-1A batches secreted ≥ 2 ng/mL/10^6^ cells of VEGF, which is our potency criteria for qualifying Stempeucel® for CLI indication (Fig. 3a). Similar to VEGF, both Stempeucel®-1 and Stempeucel®-1A also secreted substantial and comparable levels of SDF-1α (Stempeucel®-1: 568.5 ± 2.3 pg/mL/10^6^ cells; Stempeucel®-1A: 738.7 ± 24.7 pg/mL/10^6^ cells) (Fig. 3b), IL-8 (Stempeucel®-1: 13.3 ± 0.1 ng/mL/10^6^ cells; Stempeucel®-1A: 8.9 ± 0.03 ng/mL/10^6^ cells) (Fig. 3c), and TGFβ1 (Stempeucel®-1: 1.15 ± 0.01 ng/mL/10^6^ cells; Stempeucel®-1A: 1.36 ± 0.01 ng/mL/10^6^ cells) at 72 h (Fig. 3d). Significant variation in growth factor secretion is likely because of the difference in sample numbers between Stempeucel®-1 and Stempeucel®-1A. Next, the functional activity of the pooled cells was evaluated using the CM derived from three batches of Stempeucel®-1 and Stempeucel®-1A by looking at their ability to induce migration, proliferation, and endothelial tube formation in HUVEC in vitro. We observed little or no difference in the induction of HUVEC migration activity between the two versions of Stempeucel® (*P* = 0.59) and inhibition of migration was observed in the presence of an anti-VEGF mAb (Fig. 3e). Similarly, no difference was observed in the proliferation of HUVECs induced by CM obtained from the two different versions of Stempeucel® (*P* = 0.78) (Fig. 3f) and their ability to induce tube formation activity (*P* = 0.79) (Fig. 3g). Both these activities were significantly abrogated (proliferation > 95%; tube formation > 50%) [Fig. 3f; Fig. 3g (panels a, b)] by the neutralizing anti-VEGF mAb. The results obtained using positive control EGM medium and negative control medium are shown in Fig. 3e–g. The overall results presented here showed a significant correlation in the angiogenic potency of Stempeucel®-1 and Stempeucel®-1A in all three functional assays (Fig. 3). In summary, the two products manufactured from the same three donor-derived BMMSC pools demonstrated equivalent angiogenic function in vitro, in spite of observing some variation in their angiogenic cytokine quantities.

### Stempeucel® administration improves limb functionality and protects from foot necrosis in a mouse model of limb ischemia

**Fig. 4:**
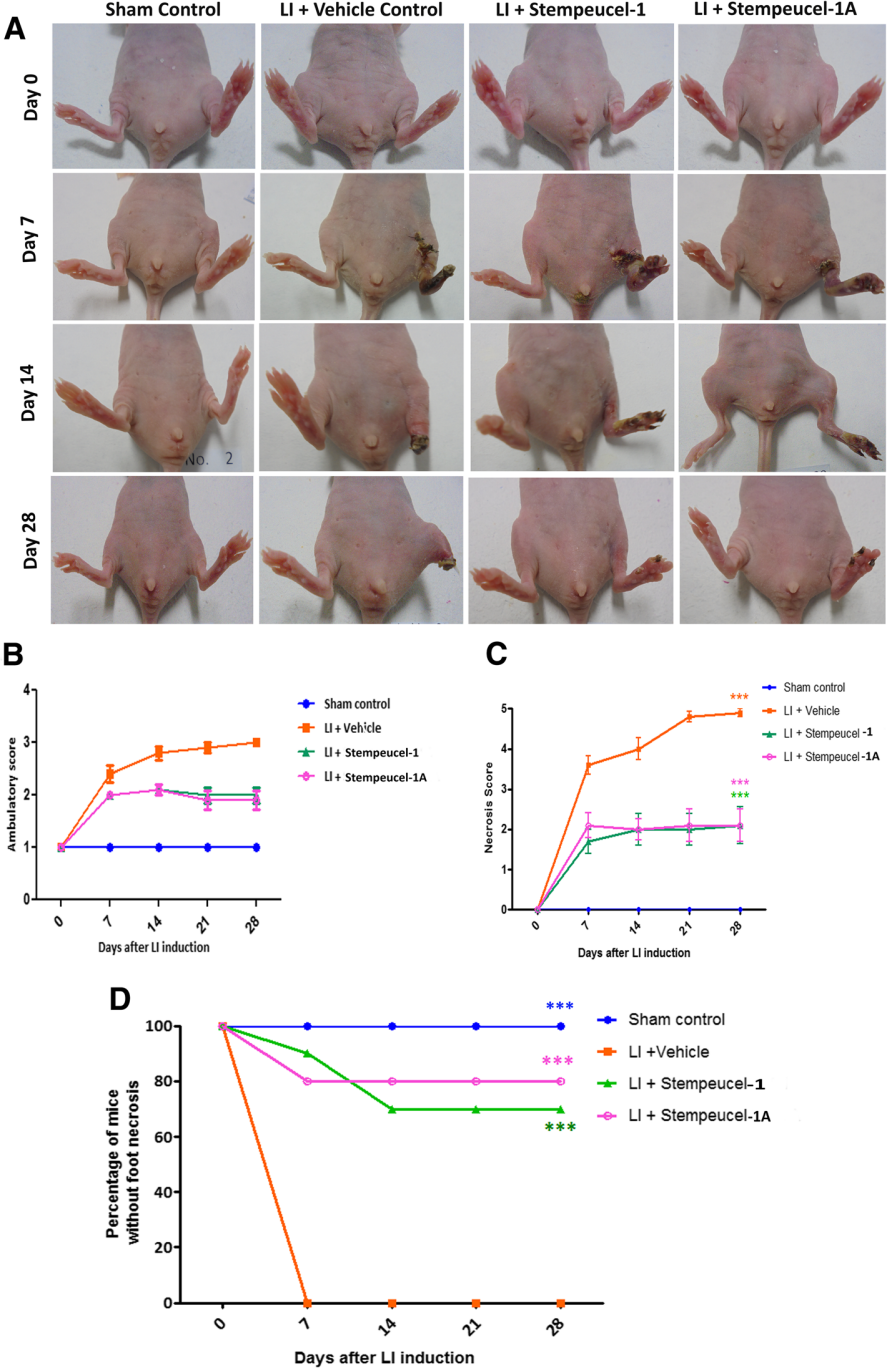
**a** Stempeucel® improves limb functionality and protects from foot necrosis in HLI mouse model: **a** Treatment with both Stempeucel®-1 and Stempeucel®-1A can salvage limbs in HLI BALB/c mouse model. Representative photographs of sham control, vehicle control, and Stempeucel®-treated animals from day 0 to 28 are shown. Stempeucel® improves limb functionality and protects from foot necrosis in HLI mouse model: **b** Mean ambulatory and; **c** necrotic score in sham control, vehicle control, Stempeucel®-1- and Stempeucel®-1A-treated animals at different time points; necrotic score-Stempeucel®-1 and Stempeucel®-1A ***P < 0.001 versus the vehicle control group. **d** Percentage of mice completely protected from foot necrosis in sham control, vehicle control, and Stempeucel®-1- and Stempeucel®-1A-treated animals at different time points; Stempeucel®-1- ***P < 0.001, Stempeucel®-1A- ***P < 0.001 versus vehicle-injected group

As described previously [13], limb necrosis and muscle damage were clearly observed on day 7 after ischemia induction in the vehicle control and the two Stempeucel® treatment groups. Auto-amputation was evident in the vehicle control group on day 14 (Fig. 4a). In fact, 70–80% of animals treated with Stempeucel®-1 and Stempeucel®-1A showed marked protection of limbs from ischemia and only minor toe necrosis was noticed on day 28 (Fig. 4a, d). However, two animals in Stempeucel®-1 treatment group and one animal in Stempeucel®-1A group did show foot necrosis (Supplementary Table 4). Significant improvement was observed in limb function (Fig. 4b), and protection from limb necrosis (Fig. 4c) was comparable between the animals receiving Stempeucel®-1 and Stempeucel®-1A. Moreover, these functional and pathological changes observed with the vehicle control animals significantly improved when the animals were treated with either Stempeucel®-1 or Stempeucel®-1A (Fig. 4b, c) with no noticeable difference observed between the two cell groups. Gross assessment of ischemic severity revealed that 90% of the animals in the vehicle treatment group underwent spontaneous limb amputation and one animal had severe foot necrosis. In contrast, both Stempeucel®-injected groups demonstrated significant limb repair and salvage (Supplementary Table 4). The majority of BMMSC-treated animals showed toe necrosis only, and percentage of animals free of foot necrosis was 70% for Stempeucel®-1 and 80% for Stempeucel®-1A respectively (Fig. 4d; Supplementary Table 4). The functional equivalence shown by the two versions of Stempeucel® did not change with the change made in the cryopreservation solutions.

### Stempeucel® treatment improves ischemia-induced muscle regeneration and recovers blood flow in the ischemic limbs

**Fig. 5:**
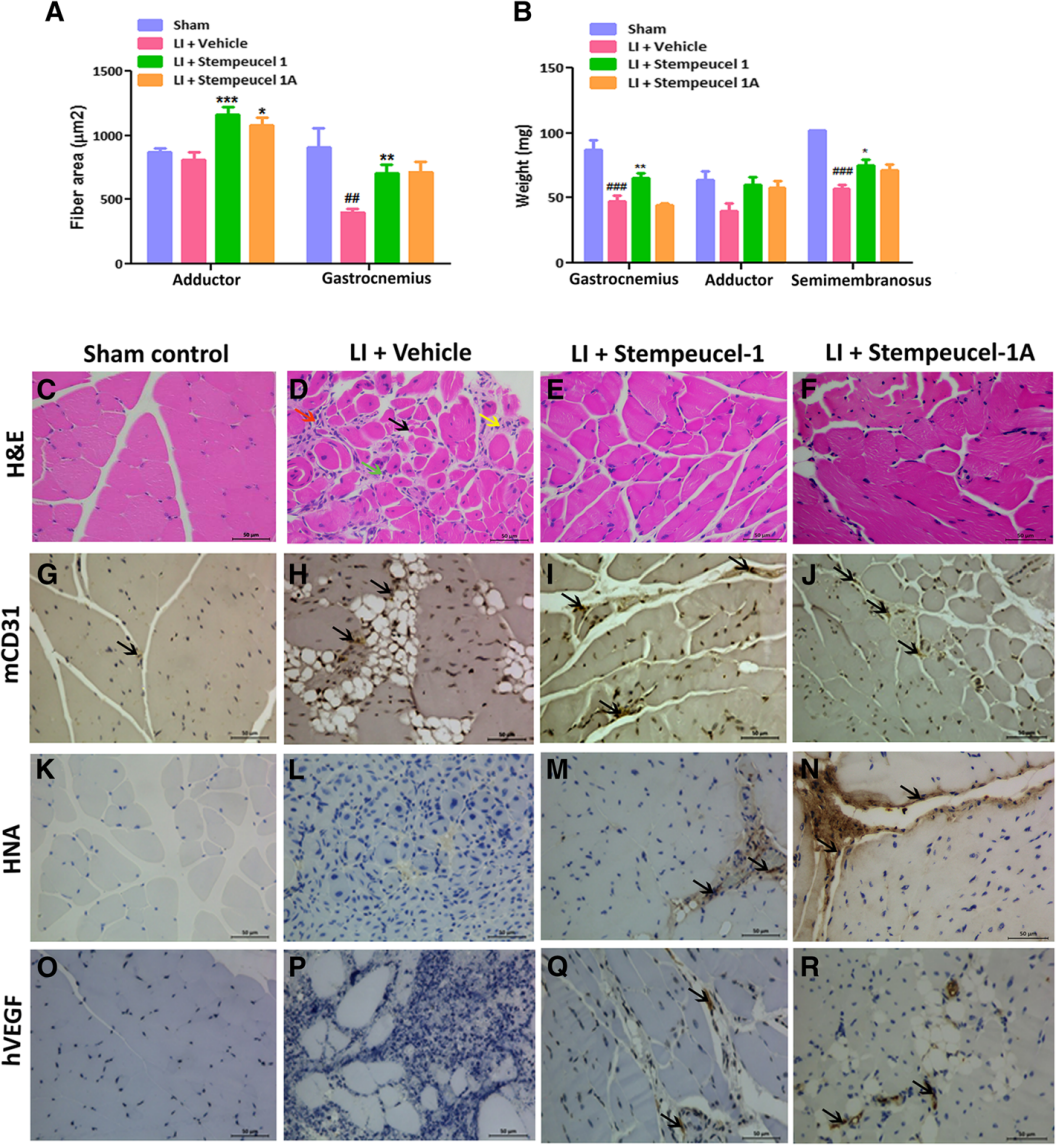
A similar magnitude of reduction in muscle degeneration and improvement in angiogenesis in the ischemic limbs of mice following Stempeucel®-1 and Stempeucel®-1A treatment: **a** Treatment with Stempeucel®-1 and Stempeucel®-1A significantly protected muscle fiber loss of adductor and gastrocnemius muscles compared to vehicle control. **b** Protection from muscle weight loss was observed upon treatment with Stempeucel®-1 and Stempeucel®-1A compared to the vehicle-treated group. **c–f** Histological analysis of muscle section of the sham control, vehicle control, and Stempeucel-treated groups. Red arrow indicates vacuolar degeneration, green arrow—mononuclear cells infiltration. Yellow arrow—muscle necrosis and black arrow—muscle degeneration. Intramuscular administrations of Stempeucel®-1 and Stempeucel®-1A survive in ischemic limb tissue, secrete paracrine factors, and recruit or proliferate CD31 positive endothelial cells. Representative images of IHC staining for **g–j** mCD31; **k–n** HNA; **o–r** hVEGF; positive cells in limb muscles of sham control, vehicle control, and Stempeucel-treated animals at day 28. Positive areas are marked with black arrows. Photographs were taken at × 40 magnification, and the scale bar shows the 50 μm

In general, we noticed a significant decrease in the muscle fiber area (Fig. 5a) and muscle weight (Fig. 5b) of LI animals treated with the vehicle. Treatment with Stempeucel®-1 and Stempeucel®-1A offered significant prevention of muscle fiber loss due to ischemia in both adductor and gastrocnemius muscles (Fig. 5a). Similarly, a significant loss in muscle weight was seen in animals treated with vehicle (adductor:40 ± 6.2 mg and gastrocnemius: 47 ± 4.5 mg), compared to the sham control group of animals (adductor: 64 ± 6.9 mg and gastrocnemius: 87 ± 7.8 mg). Substantial prevention of muscle weight loss was observed in the animals treated with Stempeucel®-1 (adductor: 60 ± 6.3 mg and gastrocnemius: 58 ± 5 mg) and Stempeucel®-1A (adductor: 65 ± 3.9 mg and gastrocnemius: 44 ± 2 mg) compared to the vehicle control group (Fig. 5b). It should be noted that we observed a difference between the weights of gastrocnemius muscle taken from Stempeucel®-1 and Stempeucel®-1A groups of animals. Although the reason for such a difference is not clear, it might have been due to incomplete administration of Stempeucel®-1A in the gastrocnemius muscle. Similar difference was not observed when the two BMMSC cell types were injected into the adductor and semimembranosus muscles (Fig. 5b). We also did not see a significant difference in muscle fiber area (Fig. 5a) between animals treated with Stempeucel®-1 or Stempeucel®-1A. The variation observed in gastrocnemius muscle weight between the two cell groups is likely because of the smaller size of the gastrocnemius muscle (Fig. 5b). In addition, no significant difference was observed in the muscle fiber area (Fig. 5a) or muscle weight (Fig. 5b) of animals treated with Stempeucel®-1 and Stempeucel®-1A. Administration of both the Stempeucel® products prevented aggravated muscle atrophy induced by femoral artery ligation, suggesting that pooled BMMSCs induced muscle regeneration in the affected limbs of the treated animals. As expected, sham control animals did not exhibit muscle degeneration and necrosis (Fig. 5c). We also observed extensive inflammation, degeneration, and necrosis in animals treated with vehicle (Fig. 5d) compared to those in the sham group (Fig. 5c) of animals but not in the other two groups receiving Stempeucel®-1 and Stempeucel®-1A (Fig. 5e, f).

A significant increase in the total mean histology scores (inflammation, degeneration, and necrosis) was also observed with adductor, soleus, gastrocnemius, and semimembranous muscle in the vehicle-treated LI animals (Fig. 5c) when compared to the sham control group (Fig. 5d) (**P < 0.001). Importantly, Stempeucel®-1- and Stempeucel®-1A-treated animals also showed significant improvement in all aspects of muscle regeneration and reduction of inflammation (Fig. 5e, f) (**P < 0.001) when compared to the animals treated with the vehicle (Fig. 5c). Further, we observed that muscle tissue from Stempeucel®-treated animals had a significant increase in the number of mouse CD31^+^ cells (Fig. 5i, j), which indicates an increase in the capillary density and arteriogenesis or angiogenesis in all muscles compared to the control group (*p* < 0.01) (Fig. 5g, j). Also, the number of CD31^+^ vessels within the muscle fibers were markedly increased in mice injected with both versions of Stempeucel® compared to the vehicle-treated animals (Fig. 5g–j). Engraftment of Stempeucel® was confirmed by positive immunohistochemical staining with human nuclear antigen (HNA) (Fig. 5k–n) and human VEGF (Fig. 5o–r) in the ischemic tissues of treated animals (Fig. 5k–n). As expected, the sham control and vehicle-treated muscles were devoid of HNA (Fig. 5k, l) and hVEGF (Fig. 5o, p) positivity while both HNA (Fig. 5m, n) and hVEGF (Fig. 5q, r)-positive cells were detected only in the muscles injected with the two versions of Stempeucel®.

**Fig. 6:**
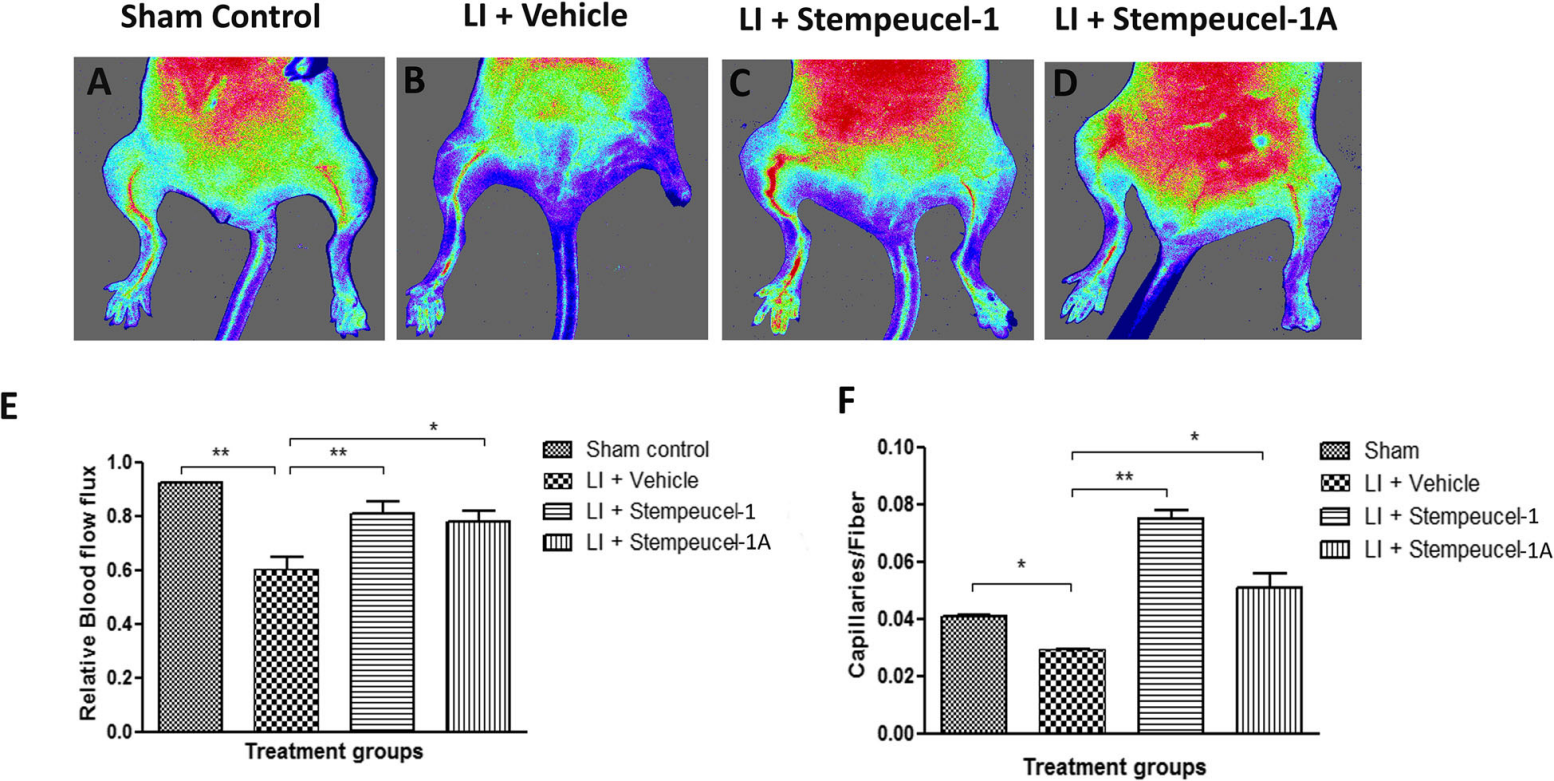
Stempeucel®-1 and Stempeucel®-1A treatment improved blood perfusion and capillary density in ischemic limbs. **a, b** Laser Doppler blood flow flux was measured in sham, LI + vehicle, and LI + two Stempeucel®-treated groups at 28 days after LI. ##*P* < 0.01, sham control vs LI vehicle; **P < 0.01, LI vehicle vs LI + Stempeucel®-1; *P < 0.05, LI vehicle vs LI + Stempeucel®-1A. **c** Capillary density at day 28 increased significantly in Stempeucel®-1- (**P < 0.001) and Stempeucel®-1A (**P < 0.01)-treated animals compared to vehicle-treated animals. Values are expressed as mean ± SEM. Scale bar = 50 μm, × 40 magnification

On day 28, normal blood flow was first observed in hind limbs of sham control animals by laser Doppler perfusion imaging (LDPI) (Fig. 6a) but not in vehicle-treated animals (Fig. 6b). Importantly, almost complete recovery of microvascular blood flow in all five mice injected with Stempeucel®-1 (*P* < 0.01) (Fig. 6c, e) and Stempeucel®-1A (*P* < 0.01) (Fig. 6d, e) was also observed while ischemic limbs of vehicle control animals showed a darker color with leg necrosis (Fig. 6b, e). Recovery of blood flow, measured by flow flux in the Stempeucel® product-administered animals, correlated well with increased capillary density (Fig. 6f), suggesting that the MoA of Stempeucel® is through an increase in neoangiogenesis and arteriogenesis in the ischemic muscles (Fig. 6).

### Stempeucel® is predominantly localized in the muscle area of ischemic limbs

**Fig. 7:**
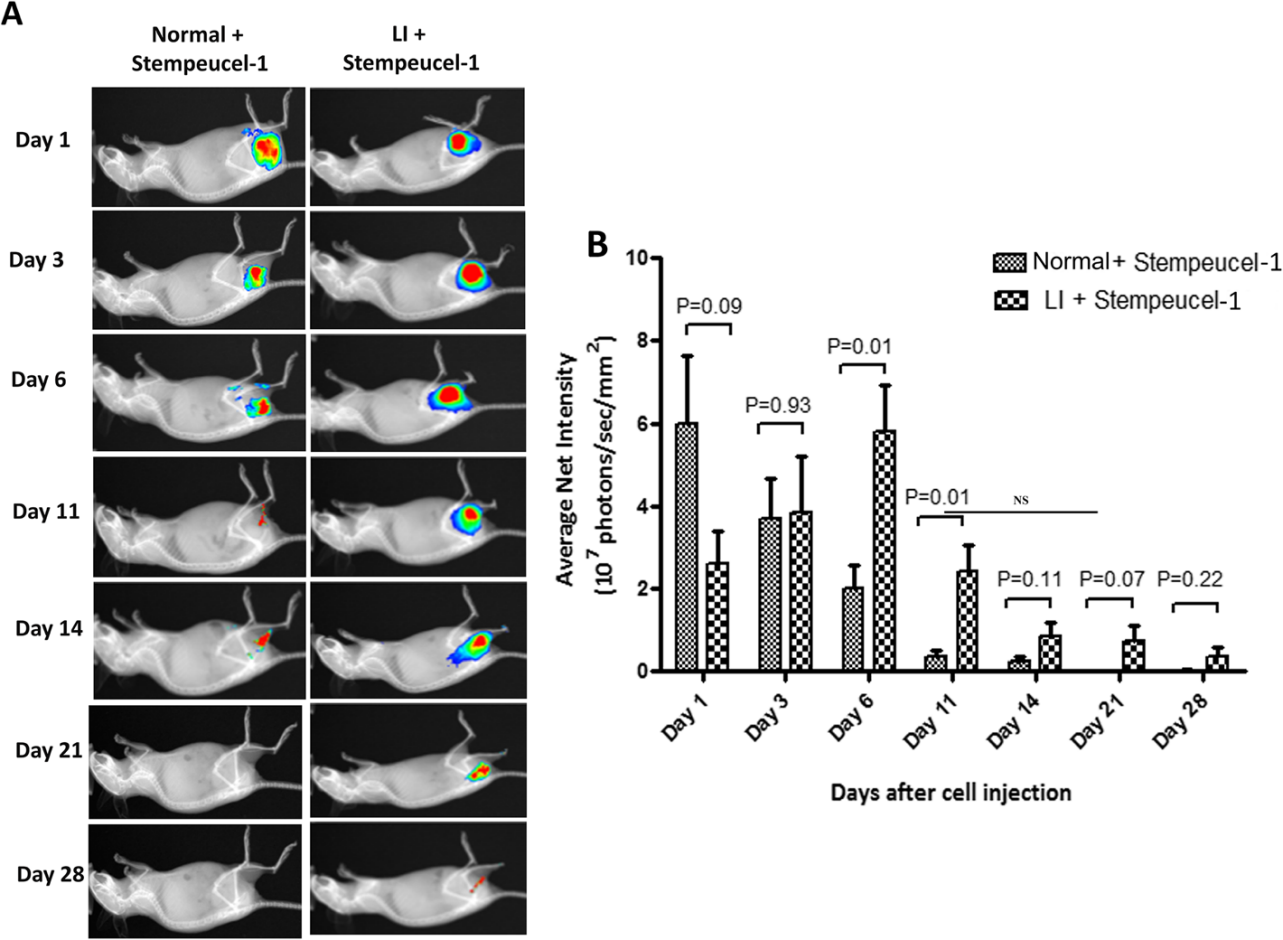
Biodistribution kinetics of Stempeucel®-1 in sham and ischemia-induced BALB/c nude mice. **a** Heat map of DiI-labeled areas in the limb muscle of sham control and ischemic animals (one representative animal shown) at various time points after Stempeucel®-1 injection. RGB scale—1 × 10^7^–10 × 10^7^ photons/s/mm^2^. **b** Graphical representation of the average net intensity of DiI-labeled cells in the limb muscles of sham and ischemic animal groups on day 1, day 3, day 6, day 11, day 14, day 21, and day 28 (end of the experiment). Significantly higher intensity of DiI signal was observed in the ischemia-induced animal group on day 6 and 11 (*P = 0.01) compared to that of the sham group

Overall, the biodistribution pattern of Stempeucel®-1 showed that intramuscularly injected cells persisted only at the injected muscle site for the study duration of 28 days and did not get distributed to other organs in detectable intensity in normal or ischemic animals (Fig. 7a). However, signal intensity kinetics revealed differences in the magnitude of the signal in non-ischemic vs. ischemic animals during the study course. The average signal intensity peaked at day 1 in normal animals (*n* = 6), whereas, in ischemic animals (*n* = 6), the intensity was approximately 50% lower compared to the control mice at the same time point. While the intensity of the DiI signal from the administered cells in normal mice started to decline rapidly from day 3 onwards, the DiI signal was found to increase in ischemic animals at day 3 and finally peaked at day 6 (Fig. 7b). The DiI signal continued to decline in all control animals progressively from day 3. No or low signal intensity was detected on days 11 and 14 and practically disappeared at the later time points. In contrast, in all ischemic animals, the DiI signal was significantly higher at day 6 (*P = 0.01) and day 11 (*P = 0.01) compared to the non-ischemic animals. The signal intensity remained slightly higher in the ischemic animals compared to the control group for the remainder of the experiment (Fig. 7b). Taken together, these data demonstrate that the biodistribution of BMMSC in ischemic animals is kinetically different from that of non-ischemic, normal animals. Since Stempeucel®-1 was slated to be used in clinical trials, the biodistribution study was only performed with this first-generation product.

## Discussion

Cell therapy has been established to be a novel therapeutic approach to treat several degenerative diseases, including CLI, for which none or few treatment options are available. During the past decade or so, MSCs from various tissue sources have proven beneficial in vascular remodeling in ischemic diseases as well as wound healing [5, 25, 26]. The primary MoA is believed to be operative with MSCs through the secretion of paracrine factors by these cells, which can deliver therapeutic signals required to repair and restore the architecture and function of damaged tissues.

Some of the important recommendations of a complex biological cell therapy product are identity, purity, potency, and safety of the therapeutic cellular products. According to the FDA requirements, comparability between various batches of products generated at different time points from one or more tissue donors must be established by both in vitro and in vivo parameters to maintain continuity of the product through validation for a specific disease indication [27]. Therefore, we performed these extensive comparative studies using Stempeucel® manufactured from two different WCBs generated from the MCBs of the same three qualified BM donors at an interval of 2 years. These studies were specifically undertaken to determine the robustness of our manufacturing process and ensure that the pooled BMMSC product, derived from the same donors using our novel technology platform, retain similar functional efficacy at different time intervals. This is one of the first studies where the comparability and consistency of GMP manufactured products from two different batches of pooled BMMSC-containing WCBs have been demonstrated in terms of their therapeutic potential to repair the damage. Global transcriptional profiling of Stempeucel® batches from the two different WCB lots (WCB-1 and WCB-1A) did not reveal any significant differences in gene expression profile, demonstrating none or little heterogeneity between the two WCBs (data not shown).Although we cannot completely rule out the functional influence of the differentially expressed genes, we did not observe substantial differences in their functional immunomodulatory and angiogenic attributes in vitro and in vivo. Additionally, we confirmed that the expression levels were comparable for angiogenic and immunomodulatory genes in both Stempeucel®-1 and Stempeucel®-1A by microarray and RT-PCR, and the results indeed showed a positive correlation for the selected genes (Fig. 1).

Different batches of Stempeucel® manufactured from the two WCB lots secreted moderate to high levels of key angiogenic factors, namely, VEGF, SDF-1α, IL-8, and TGFβ1. Importantly, both Stempeucel®-1 and Stempeucel®-1A secreted VEGF at a range of 2–5 ng/mL/10^6^ cells, which has been determined to be the range of VEGF necessary to qualify every batch of Stempeucel® product [17]. Furthermore, in functional angiogenic experiments, Stempeucel®-1 and Stempeucel®-1A, showed similar activity of promoting migration, proliferation, and tube formation of HUVECs in vitro. Similarly, another group of investigators demonstrated that VEGF is the key molecule alongside IL-8 and CXCL-5 secreted by Multistem® to promote angiogenic functions in vitro and in vivo [28]*.*Stempeucel®-1 and Stempeucel®-1A were found to have highly comparable immunomodulatory activity and this activity was found to be dose-dependent. Mimicking the exposure of Stempeucel®-1 and Stempeucel®-1A to an inflammatory environment in vitro, by exposure to IFN-γ and TNFα, resulted in similar upregulation of HLA-DR in both versions of Stempeucel®. HLA-DR upregulation has also been linked to the increased expression of IDO and PGE-2, and these molecules are known to suppress T cell effector function [29, 30]. Recently published literature suggests that MSCs isolated from various tissues, when licensed with IFN-γ, effectively suppress PHA stimulated T cell proliferation and protect themselves from T cell-mediated apoptosis [31]. Consistent with the published literature [24], we found that INFγ and TNFα licensing of Stempeucel®-1 and Stempeucel®-1A rendered these pooled BMMSCs with augmented allogeneic MLR suppressive activity compared to their unprimed counterparts (Fig. 3).

We have earlier determined that a cell dose of 5 × 10^6^ of Stempeucel®-1 is optimal to repair ischemic limbs in mice; hence, we chose to use the same dose to demonstrate product comparability between the two versions of Stempeucel in the same mouse model [7, 13]. The results clearly showed significant improvements in limb function, foot and leg necrosis, and leading to complete limb salvage in a few animals in both groups. These results were further substantiated by the fact that the administration of these cells completely restored the blood flow in the ischemia-induced animals by 28 days post cell administration. When Norgren et al. injected 1 × 10^6^ placenta-derived MSCs (PLX-PAD) into the ischemic limb muscles of mice, they observed ≥ 70% recovery of blood perfusion into the limbs, which were significantly greater than that of the placebo at 35 days [32]. Similarly, a dose-dependent recovery of blood blow was observed upon injecting human neural stem cells (hNSCs) into the ischemic limbs of immunocompetent CD-1 mice, although significant recovery was seen with 3 × 10^5^ cells/muscle dose group [33]. Although a direct comparison of our data and the data obtained by PLX-PAD and hNSCs is not possible, it appears that the extent of blood flow throughout the limb with Stempeucel® was higher (as judged by the pathologist to be a complete recovery of blood flow). Overall, the in vitro and in vivo functional data generated with Stempeucel®-1 and Stempeucel®-1A demonstrated that the two pooled products are highly comparable.

Interestingly, the biodistribution analysis showed that Stempeucel®-1 was mainly localized at the injection site, in the muscle of both normal and ischemic mice (Fig. 7); however, the kinetics of cellular distribution at the injected site varied significantly between normal and ischemic animals. This difference is likely due to the disease state of ischemic muscles, where the cellular localization peaked at day 6. In contrast, the average signal intensity peaked in the normal animals within 24 h of administration (Fig. 7). Though the exact reason is not clear, it may be possible that sudden exposure of the BMMSC to the severe ischemic microenvironment might have shunted cell proliferation or induced cell death at the injected tissue site. The surviving cells subsequently proliferated in order to repair the damage caused in the ischemic limb. Intramuscularly injected Stempeucel® persisted only at the site of injection for 28 days and was not distributed to other organs in both ischemic and normal animals. Although it is difficult to explain the reason for such differences in the kinetics of maximum DiI signal intensity between the two groups, it is enticing to speculate that Stempeucel® stayed longer in ischemic mice until the inflammation subsided and the affected muscles were repaired by blood flow and neoangiogenesis.

## Conclusion

One of the main objectives of this study was to determine the functionality of Stempeucel® obtained from two independently derived cell stocks, produced at different times. The entire comparative study centered around the hypothesis that it is possible to extend the lifespan of the product with comparable in vitro and in vivo properties once the MCB stock and the manufacturing WCB lots are consumed to a large extent by creating a second set of cryopreserved MCBs and WCBs. In order to demonstrate that the pooled BMMSC products obtained from these two versions of WCBs were highly comparable for gene and protein expression important for regulating angiogenesis and repairing ischemia-induced damage in the limb tissue, we conducted a series of studies and established equivalency between the two pooled products that were manufactured from the same three healthy individuals whose BM tissue was aspirated at two different time points. These observations led us to conclude that pooled cell therapy products of equivalent efficacy can be produced through a robust GMP manufacturing process at different times. Having established that the lifetime of a product can be extended for a considerable period using cell pooling technology, our future aim would be to develop and manufacture the next product that would use BM from three new human donors. Whether certain additional criteria of individual donor-derived BMMSC such as proliferation capacity, angiogenic and anti-inflammatory abilities would enhance the therapeutic potential of a pooled BMMSC product is open to experimentation.

## Supplementary Information

**Additional file 1: Supplementary Table 1.** Characterization of Stempeucel®-1 and 1A.

**Additional file 2: Supplementary Table 2.** List of angiogenesis-related genes.

**Additional file 3: Supplementary Table 3.** List of inflammation-related genes.

**Additional file 4: Supplementary Table 4.** Amelioration of Necrosis and limb salvage by intramuscular administration of Stempeucel®-1 and 1A in a BALB/c nude mouse model of Hind Limb Ischemia.

## Data Availability

All data generated or analyzed are included in this research article.

## Abbreviations

ABI: Ankle-brachial index
ACP5: Acid Phosphatase 5, Tartrate resistant
ADSC: Adipose tissue-derived mesenchymal stromal cells
AGAP: ArfGAP with GTPase Domain
AHI1: Abelson Helper Integration Site 1
Ang-1: Angiopoietin-1
BM: Bone marrow
BMMNC: Bone marrow mononuclear cells
BMMSC: Bone marrow-derived mesenchymal stromal cells
BrdU: Bromodeoxyuridine
CLI: Critical limb ischemia
CM: Conditioned medium
CNTNAP3: Contactin associated protein family member 3
CS5: CryoStor 5
CXCR1: C-X-C-motif chemokine receptor 1
ELISA: Enzyme-linked immunosorbant assay
EPC: Endothelial progenitor cells
GMP: Good Manufacturing Practice
HFF: Human foreskin fibroblast
HGF: Hepatocyte growth factor
HLA-DR: Human leukocyte antigen DR
HLI: Hind limb ischemia
HNA: Human nuclear antigen
hNSC: Human neural stem cells
HSA: Human serum albumin
HSC: Hematopoietic stem cells
HUVEC: Human umbilical vein endothelial cells
i.m: Intramuscular
IDO: Indoleamine 2,3-dioxygenase
IFN-γ: Interferon-γ
IL-6: Interleukin 6
IL-8: Interleukin 8
IVIS: In vivo imaging system
LAMA1: Laminin subunit alpha 1
LDPI: Laser Doppler perfusion imaging
LI: Limb ischemia
MCB: Master cell bank
MEF2C: Myocyte enhancer factor 2C
MLR: Mixed lymphocyte reaction
MoA: Mechanism of action
MPC: Mesenchymal progenitor cells
MSC: Mesenchymal stromal cells
PAD: Peripheral arterial disease
PDL-1: Programmed death ligand 1
PDMSC: Placenta-derived mesenchymal stromal cells
PID 1: Phosphotyrosine interaction domain containing 1
PLX: Placenta-derived stromal cell product
PTGDR: Prostaglandin reductase
SDF-1α: Stromal cells derived factor 1α
SEM: Standard error of mean
TcPO_2_: Transcutaneous oxygen pressure
TGFβ1: Transforming growth factor β1
TNFα: Tumor necrosis factor α
UCHL1: Ubiquitin carboxy-terminal hydrolase L1
VCAM: Vascular cell adhesion molecule
VEGF: Vascular endothelial growth factor
WCB: Working cell banks
WJ-MSC: Wharton’s jelly-derived mesenchymal stromal cells
ZEB1: Zinc finger E-box binding homeobox 1
