# Supplementary material for: Human bone marrow-derived, pooled, allogeneic mesenchymal stromal cells manufactured from multiple donors at different times show comparable biological functions in vitro, and in vivo to repair limb ischemia
Source: Stem Cell Res Ther. 2021 May 10;12:279. doi: 10.1186/s13287-021-02330-9 (PMC8108338; doi:10.1186/s13287-021-02330-9)
Supplement: Supplementary file 1 — Additional file 1: Supplementary Table 1. Characterization of Stempeucel®-1 and 1A. [file 13287_2021_2330_MOESM1_ESM.docx]

**Supplementary Table 1: Characterization of Stempeucel®-1 and 1A**

| **Stempeucel®-1** | | | | | **Stempeucel®-1A** | | | | |
| --- | --- | --- | --- | --- | --- | --- | --- | --- | --- |
| **Percentage of cells positive for MSC markers (%)** | **Percentage of cells negative for MSC markers (%)** | **Percentage of cells expressing co-stimulatory markers (%)** | **Cell Viability (%)** | **Differentiation** | **Percentage of cells positive for MSC markers (%)** | **Percentage of cells negative for MSC markers (%)** | **Percentage of cells expressing co-stimulatory markers (%)** | **Cell Viability**  **(%)** | **Differentiation** |
| CD44 100±0  CD73 100±0  CD90 97±1.5  CD105 99±0.5  CD166 100±0  HLA-ABC 97±1 | HLA-DR 18±4.5  CD34 0.3±0.5  CD45 0±0 | CD40 1.3±0.5  CD80 1.2±0.5  CD86 2±1.7 | ≥90% | Positive for Adipogenesis, Osteogenesis and chondrogenesis | CD44 100±0  CD73 100±0  CD90 92.6±5.8  CD105 99.2±0.3  CD166 100±0  HLA-ABC 95.9±0.9 | HLA-DR 8±3  CD34 0±0  CD45 0±0 | CD40 0.7±0.4  CD80 1.9±2.15  CD86 1.5±2.1 | ≥90% | Positive for Adipogenesis, Osteogenesis and chondrogenesis |

Table 1.1: Data from three Stempeucel®1 and 1A are presented. Values are represented as mean ± SEM. SEM, Standard Error of Mean; MSC, Mesenchymal stromal cells; PCR, Polymerase chain reaction; HLA, Human leukocyte antigen D-related; Stempeucel®1 and 1A were found to be negative for endotoxin content (<0.06 endotoxin units/mL), negative for mycoplasma by PCR, karyotyping was found to be normal, 46 XY, and showed normal DNA ploidy index (*data not shown)

**Table 1.2. Antibodies used for flow cytometry analysis**

| **Antibody** | **Manufacturer** | **Cat No.** | **Conjugate** | **Stock Concentration** | **Dilution** |
| --- | --- | --- | --- | --- | --- |
| ISO control PE | BD Pharmingen | 550617 | PE | 200 μg/ml | 1:16 |
| ISO control FITC | BD Pharmingen | 550616 | FITC | 500 μg/ml | 1:16 |
| ISO control FITC | BD Pharmingen | 555573 | FITC | 50 μg/ml | 1:2 |
| CD44 | BD Pharmingen | 550989 | PE | 12.5 μg/ml | Neat |
| CD73 | BD Pharmingen | 550257 | PE | 6.25 μg/ml | Neat |
| CD90 | BD Pharmingen | 555596 | PE | 200 μg/ml | Neat |
| CD105 | R&D systems | FAB10971P | PE | 25 μg/ml | Neat |
| CD166 | BD Pharmingen | 559263 | PE | 12.5 μg/ml | Neat |
| HLA-ABC | BD Pharmingen | 555555 | APC | 3 μg/ml | Neat |
| CD34 | BD Pharmingen | 550761 | PE | 12.5 μg/ml | Neat |
| HLA-DR | BD Pharmingen | 347363 | FITC | 25 μg/ml | 1:50 |
| CD40 | BD Pharmingen | 560963 | PE | 12.5 μg/ml | Neat |
| CD80 | BD Pharmingen | 340294 | PE | 0.0032 mg/ml | Neat |
| CD86 | Biolegend | 305405 | PE | 0.2 mg/ml | Neat |
