# Supplementary material for: Human bone marrow-derived, pooled, allogeneic mesenchymal stromal cells manufactured from multiple donors at different times show comparable biological functions in vitro, and in vivo to repair limb ischemia
Source: Stem Cell Res Ther. 2021 May 10;12:279. doi: 10.1186/s13287-021-02330-9 (PMC8108338; doi:10.1186/s13287-021-02330-9)
Supplement: Supplementary file 2 — Additional file 2: Supplementary Table 2. List of angiogenesis-related genes. [file 13287_2021_2330_MOESM2_ESM.docx]

**Supplementary Table 2:**

**List of angiogenesis-related genes**

| Gene symbol | Name | Stempeucel®-1 vs HFF | Stempeucel®-1A vs HFF |
| --- | --- | --- | --- |
| VEGFA | vascular endothelial growth factor A | 2.27 | 2.38 |
| PDGFC | Platelet Derived Growth Factor C | 2.78 | 2.82 |
| TGF beta 1 | Transforming growth factor beta 1 | 2.01 | 6.76 |
| TGF beta 2 | Transforming growth factor beta 2 | 11.13 | 9.99 |
| TGF beta 3 | Transforming growth factor beta 3 | 3.2 | 3.74 |
| IGF-1 | Insulin like growth factor 1 | 2.08 | 2 |
| PDGFRa | Platelet Derived Growth Factor receptor a | 3.17 | 3.06 |
| HGF | Hepatocyte growth factor | 3.05 | 2.68 |
| EDN1 | Endoglin | 2.57 | 2.75 |
| CXCL8 | Chemokine (C-X-C Motif) Ligand 8 | 3.99 | 4.1 |
| EPHA2 | Epithelial Cell Receptor Protein Tyrosine Kinase | 2.66 | 2.49 |
| EPHB2 | Epithelial Cell Receptor Protein Tyrosine Kinase | 3.92 | 3.86 |
| MMP1 | Matrix metallo proteinase1 | 7.6 | 4.7 |
| VCAM | vascular cell adhesion molecule 1 | 5.36 | 2.08 |
| MMP14 | Matrix metallo proteinase14 | 2.5 | 2.13 |
| IGFBP7 | Insulin like growth factor binding protein 7 | 2.65 | 2.1 |
| ITGB3 | integrin, beta 3 | 2.56 | 3.12 |
| AGPT1 | angiopoietin 1 | 2.22 | 2.5 |
| ITGA2 | integrin, alpha 2 (CD49B, alpha 2 subunit of VLA-2 receptor) | 4.43 | 3.9 |
| ESM1 | Endothelin specific molecule-1 | 2.34 | 2.8 |
| EDIL3 | EGF-like repeats and discoidin I-like domains 3 | 6.5 | 5.23 |
| LOXL2 | lysyl oxidase-like 2 | 3.99 | 3.34 |
| ITGB5 | integrin, beta 5 | 3.02 | 2.98 |

List of angiogenic genes up-regulated (P< 0.005) in Stempeucel®-1 and 1A vs human foreskin fibroblast (HFF)
