# Supplementary material for: Human bone marrow-derived, pooled, allogeneic mesenchymal stromal cells manufactured from multiple donors at different times show comparable biological functions in vitro, and in vivo to repair limb ischemia
Source: Stem Cell Res Ther. 2021 May 10;12:279. doi: 10.1186/s13287-021-02330-9 (PMC8108338; doi:10.1186/s13287-021-02330-9)
Supplement: Supplementary file 3 — Additional file 3: Supplementary Table 3. List of inflammation-related genes. [file 13287_2021_2330_MOESM3_ESM.docx]

**Supplementary Table - 3**

**List of inflammation-related genes**

| Gene symbol | Name | Stempeucel®-1 vs HFF | Stempeucel®-1A vs HFF |
| --- | --- | --- | --- |
| IDO | Indoleamine 2,3-Dioxygenase 1 | 5.9 | 5.41 |
| IL-10 | Interleukin 10 | 5.87 | 5.01 |
| ICAM-1 | Intercellular Adhesion Molecule 1 | 4.1 | 4.8 |
| VCAM-1 | Vascular Cell Adhesion Molecule 1 | 5.6 | 5.4 |
| VEGF A | Vascular endothelial growth factor A | 2.27 | 2.38 |
| CCL2 | Chemokine (C-C Motif) Ligand 2 | 2.96 | 5.45 |
| TGF b1 | Transforming growth factor beta 1 | 2.01 | 6.76 |
| ITGB-3 | integrin, beta 3 | 2.56 | 3.12 |
| PDGF C | Platelet Derived Growth Factor C | 2.39 | 2.76 |
| CXCR-4 | Chemokine (C-X-C Motif) Receptor 4 | 2.27 | 2.12 |
| CXCR-1 | Chemokine (C-X-C Motif) Receptor 1 | 2.15 | 2.1 |
| CCR-7 | Chemokine (C-C Motif) Receptor 7 | 3.67 | 5.2 |
| FLG | Filaggrin | 6.28 | 6.8 |
| IL-8 | Interleukin 8 | 4.8 | 4.23 |
| CD-74 | CD74 Molecule, Major Histocompatibility Complex, Class II Invariant Chain | 4.43 | 4.61 |
| EDN-1 | Endothelin 1 | 2.01 | 1.99 |
| CXCL-2 | Chemokine (C-X-C motif) ligand 2 | 3.87 | 3.11 |
| HLA-G5 | HLA-G histocompatibility antigen, class I, G | 2.47 | 2.16 |
| PRDM1 | PR Domain Containing 1, With ZNF Domain | 2.54 | 2.67 |
| CX3CR1 | Chemokine (C-X3-C Motif) Receptor 1 | 3.87 | 3.33 |
| CXCR6 | Chemokine (C-X-C Motif) Receptor 6 | 1.98 | 1.05 |
| GAL1 | Galanin/GMAP Prepropeptide | 5.88 | 4.8 |
| PDL1 | CD274 Molecule | 3.09 | 2.54 |
| PDL2 | Programmed Cell Death 1 Ligand 2 | 2.76 | 2.88 |
| TSG-6 | Tumor Necrosis Factor, Alpha-Induced Protein 6 | 3.9 | 3.67 |
| BCL2 | B-Cell CLL/Lymphoma 2 | 2.78 | 2.88 |
| CHI3L1 | Chitinase 3-Like 1 (Cartilage Glycoprotein-39) | 2.58 | 2.56 |
| SLC2A1 | Solute Carrier Family 2 (Facilitated Glucose Transporter), Member 1 | 2.44 | 2.31 |
| LIF | Leukemia Inhibitory Factor | 2.05 | 2.87 |
| NEDD9 | Neural Precursor Cell Expressed, Developmentally Down-Regulated 9 | 2.13 | 2.21 |
| GLS | Glutaminase | 2.06 | 2.16 |
| PTGER2 | Prostaglandin E Receptor 2 (Subtype EP2), 53kDa | 5.34 | 5.23 |
| PRDM1 | PR Domain Containing 1, With ZNF Domain | 2.29 | 2.13 |
| CXCL12 | Chemokine (C-X-C Motif) Ligand 12 | 2.54 | 2.38 |

Immunomodulatory genes up-regulated (P< 0.005) in Stempeucel®-1 and 1A vs human foreskin fibroblast
