# Supplementary material for: Human bone marrow-derived, pooled, allogeneic mesenchymal stromal cells manufactured from multiple donors at different times show comparable biological functions in vitro, and in vivo to repair limb ischemia
Source: Stem Cell Res Ther. 2021 May 10;12:279. doi: 10.1186/s13287-021-02330-9 (PMC8108338; doi:10.1186/s13287-021-02330-9)
Supplement: Supplementary file 4 — Additional file 4: Supplementary Table 4. Amelioration of Necrosis and limb salvage by intramuscular administration of Stempeucel®-1 and 1A in a BALB/c nude mouse model of Hind Limb Ischemia. [file 13287_2021_2330_MOESM4_ESM.docx]

**Supplementary Table 4: Amelioration of Necrosis and limb salvage by intramuscular administration of Stempeucel®-1 and 1A in a BALB/c nude mouse model of Hind Limb Ischemia**

| Groups | Complete Limb Salvage^a^ | Limb Salvage with one toe necrosis^b^ | Limb Salvage with two or more toe necrosis^c^ | Limb Salvage with foot necrosis^d^ | Limb Salvage with Ankle necrosis^e^ | Limb Loss^f^ |
| --- | --- | --- | --- | --- | --- | --- |
| Sham Control  (n=3) | 100% | 0 | 0 | 0 | 0 | 0 |
| LI + Vehicle (n=10) | 0 | 0 | 0 | 0 | 10% | 90% |
| LI + Stempeucel®-1  (n=10) | 20% | 0 | 50% | 20% | 0 | 10% |
| LI + Stempeucel®-1A  (n=10) | 10% | 10% | 60% | 10% | 0 | 10% |

a- No necrosis, b- One toe necrosis, c- Two or more toe necrosis, d- Partial or complete foot necrosis, e- Necrosis in ankle and above, f- Auto-amputation of the entire leg
